# Genomic novelty within a “great speciator” revealed by a high-quality reference genome of the collared kingfisher (*Todiramphus chloris collaris*)

**DOI:** 10.1093/g3journal/jkac260

**Published:** 2022-09-26

**Authors:** Chad M Eliason, Taylor Hains, Jenna McCullough, Michael J Andersen, Shannon J Hackett

**Affiliations:** Grainger Bioinformatics Center, Field Museum of Natural History, Chicago, IL 60605, USA; Negaunee Integrative Research Center, Field Museum of Natural History, Chicago, IL 60605, USA; Department of Ecology and Evolution, Committee on Evolutionary Biology, University of Chicago, Chicago, IL 60637, USA; Department of Biology, University of New Mexico, Albuquerque, NM 87131, USA; Department of Biology, University of New Mexico, Albuquerque, NM 87131, USA; Negaunee Integrative Research Center, Field Museum of Natural History, Chicago, IL 60605, USA; Department of Ecology and Evolution, Committee on Evolutionary Biology, University of Chicago, Chicago, IL 60637, USA

**Keywords:** sensory systems, comparative genomics, Aves

## Abstract

Islands are natural laboratories for studying patterns and processes of evolution. Research on island endemic birds has revealed elevated speciation rates and rapid phenotypic evolution in several groups (e.g. white-eyes, Darwin’s finches). However, understanding the evolutionary processes behind these patterns requires an understanding of how genotypes map to novel phenotypes. To date, there are few high-quality reference genomes for species found on islands. Here, we sequence the genome of one of Ernst Mayr’s “great speciators,” the collared kingfisher (*Todiramphus chloris collaris*). Utilizing high molecular weight DNA and linked-read sequencing technology, we assembled a draft high-quality genome with highly contiguous scaffolds (scaffold N50 = 19 Mb). Based on universal single-copy orthologs, we estimated a gene space completeness of 96.6% for the draft genome assembly. The population demographic history analyses reveal a distinct pattern of contraction and expansion in population size throughout the Pleistocene. Comparative genomic analysis of gene family evolution revealed that species-specific and rapidly expanding gene families in the collared kingfisher (relative to other Coraciiformes) are mainly involved in the ErbB signaling pathway and focal adhesion. *Todiramphus* kingfishers are a species-rich group that has become a focus of speciation research. This draft genome will be a platform for future taxonomic, phylogeographic, and speciation research in the group. For example, target genes will enable testing of changes in sensory structures associated with changes in vision and taste genes across kingfishers.

## Introduction

Islands offer unique opportunities to study evolution in action in natural populations owing to their discrete geographical boundaries and well-characterized geologic histories (Losos and Ricklefs 2009). As such, islands have been key to studying several evolutionary paradigms, such as the paradox of the “great speciators” (Diamond and Mayr 1976), the Theory of Island Biogeography (MacArthur and Wilson 1967), and adaptive radiation (Losos and Ricklefs 2009). Early research focused primarily on biogeographic and phenotypic aspects of species (Wallace 1881; Mayr 1963; Mayr and Diamond 2001). More recently, researchers have begun to leverage genomic data to study evolutionary patterns and processes on islands. For example, Cornetti *et al.* (2015) studied white-eye genomes and found that this island clade harbors high rates of nucleotide substitutions, gene duplications, and positive selection relative to other bird clades. A recent study extended this work using target capture to shed light on the origins of biodiversity/lineages in white-eyes (Gwee *et al.* 2020). The phenotypes evolve rapidly on islands (Millien 2006), often in tandem with speciation rates (Cardoso and Mota 2008; Rabosky *et al.* 2013). Yet, few studies have characterized the genomes of focal clades known to show rapid rates of phenotypic evolution (e.g. Almén *et al.* 2016).

Kingfishers have rapidly evolving brains (Eliason *et al.* 2021), colorful plumages (Moyle *et al.* 2007), and diverse foraging behaviors (Woodall 1991, 2016). The collared kingfisher (*Todiramphus chloris*) is one of the most widespread of the “great speciators” (Mayr and Diamond 2001), an evolutionary lineage with an ability to rapidly form new taxa across insular geographies despite their propensity to disperse. Recently, the 50 subspecies comprising the *T. chloris* species complex, which ranged >16,000 km from the Red Sea to Polynesia (Fry 1992), were split into at least 10 different species (depending on taxonomic authority) by Andersen *et al.* (2015). This new appreciation for species-level diversity within *Todiramphus* has highlighted this genus as having elevated speciation rates relative to background rates within the order Coraciiformes (Andersen *et al.* 2018; McCullough *et al.* 2019). Recent work shows that these elevated speciation rates are associated with dispersal into island systems of Wallacea and the South Pacific (Andersen *et al.* 2018), as well as rapid rates of brain shape evolution (Eliason *et al.* 2021). However, the evolutionary processes underpinning these patterns remain unclear. For example, are changes in brain shape causally linked to the speciation process, or is speciation driven by changes in chromosomal architecture that also influence brain shape diversity as a by-product? To answer these and other questions about evolutionary processes driving speciation, a high-quality *Todiramphus* reference genome is needed.

Here, we present a high-quality draft genome of the collared kingfisher (*T. chloris collaris*). Even in its more narrowly circumscribed species limits, *T. chloris* has a wide distribution from the coasts of the Red Sea to northwestern New Guinea. Across its range, it is delineated into 14 subspecies, all with disjunct distributions. *Todiramphus chloris collari*s is an island-dwelling subspecies found throughout the Philippine Archipelago (Andersen *et al.* 2015). We further integrate these data with existing avian genomes and use a comparative genomics approach to test the hypothesis that islands are associated with genomic novelty in the collared kingfisher relative to other closely related coraciiform species.

## Materials and methods

### Genome sequencing

We used frozen liver tissue of a collared kingfisher (*T. chloris collaris*) voucher collected in 1992 from Sibuyan Island and accessioned at the Field Museum, specimen number FMNH 358326. We extracted DNA using standard protocols for high molecular weight DNA extraction (10X Genomics, CA), resulting in a concentration of 10.5 ng/µl of DNA. We constructed the library with the 10X Genomics Genome and Gel Bead Kit v2. We sequenced the genome on a paired-end 150 lane of a HiSeq 4000 at the Roy J. Carver Biotechnology Center at the University of Illinois Urbana-Champaign.

### Genome assembly

We assembled the genome using 10X Supernova (Weisenfeld *et al.* 2017) with default options. To fill gaps in the reference genome, we used the ABySS Sealer program (Paulino *et al.* 2015) with default options and 2 different kmer lengths (*k* = 64, 96). We further assessed genome completeness using BUSCO v. 5 (Manni *et al.* 2021) and by aligning our draft genome assembly to 2 related species in the Afroaves clade with high-quality chromosome-level assemblies available on GenBank (Supplementary Table 1): Abyssinian ground-hornbill (*Bucorvus abyssinicus*) and a more closely related coraciiform species, northern carmine bee-eater (*Merops nubicus*). We then used the JupiterPlot pipeline (code available at https://github.com/JustinChu/JupiterPlot; accessed 2022 September 26) to visualize scaffold sizes and synteny.

### Repeat annotation

We used RepeatModeler v. 1.0.11 (Smit and Hubley 2008) to identify repetitive elements within the genome based on the NCBI database. We located 172 repeat families and used this as an input for our downstream genome annotation pipeline (see below). We further masked repeats using this de novo set of annotations with RepeatMasker v. 4.0.1 (Smit *et al.* 2013), in addition to those for the chicken (using the “-species = chicken” argument in RepeatMasker). More than 98% of the repeats in chicken were also identified de novo in the collared kingfisher using RepeatModeler, with 21% novel repeat content in the collared kingfisher relative to chicken.

### Gene annotation

We used ortholog-based annotation implemented in GeMoMa (Keilwagen *et al.* 2018). We used 4 high-quality genome annotations to infer genes in the collared kingfisher: budgerigar (*Melopsittacus undulatus*), Anna’s hummingbird (*Calypte anna*), golden eagle (*Aquila chrysaetos*), and chicken (*Gallus gallus*) (see Supplementary Table 1). These species were chosen based on availability of a high-quality annotation as well as their phylogenetic proximity to kingfishers. Finally, we filtered predictions using the GAF function, resulting in a final protein dataset (GFF file) of 16,539 genes with an average length of 17.7 kb and a BUSCO score of 93.0% [(S: 74.8%, D: 18.2%), F: 1.0%, M: 6.0%, *n* = 8,338]. To functionally annotate this set of putative protein transcripts, we used the rbh function in mmseqs2 (Steinegger and Söding 2017) to blast to the uniprot set of genes (The UniProt Consortium 2021), converted uniprot IDs to gene names, and used this set of gene names in downstream enrichment analyses (see below).

### Population demographic history

We aligned cleaned reads to the reference genome using BWA-MEM (Li 2013). We used pairwise sequentially Markovian coalescent (PSMC) v. 0.6.5 (Li and Durbin 2011) to infer population demographic history using parameters from a previous study on bird genomes (Nadachowska-Brzyska *et al.* 2015). To plot effective population sizes (Ne) as a function of time, we used published R code (Liu and Hansen 2016) to input data into R with a generation time of 4.8 years (IUCN 2021) and a mutation rate of 2.23 × 10^−8^ mutations per generation. This mutation rate estimate was based on a molecular substitution rate of 0.11 mutations per million years for *Alcedo atthis* (Lanfear *et al.* 2010), a branch length of 24 My for this lineage (Prum *et al.* 2015), and a generation time of ∼5 years. We accounted for uncertainty by running 100 bootstrap PSMC estimates based on resampling of 100-bp genomic regions (Li and Durbin 2011).

### Protein evolution

We used OrthoFinder v. 2.5.2 (Emms and Kelly 2019) to infer gene families and CAFE v. 4.2.1 (Han *et al.* 2013) to uncover trends in gene family evolution and test our hypothesis that the collared kingfisher has elevated genomic novelty associated with island-living. We used curated genome annotations (Feng *et al.* 2020) for 4 coraciiform species (*Halcyon senegalensis*, *Chloroceryle aenea*, *Ceyx cyanopectus*, and *Todus mexicanus*) as well as 2 outgroups (*Taeniopygia guttata* and *C. anna*) (see Supplementary Table 1 for details). Outgroup species were added to the coraciiform timetree (McCullough *et al.* 2019) based on divergence estimates in Prum *et al.* (2015). We attempted to account for gene annotation error using the caferror.py function in CAFE. This algorithm optimizes likelihoods by estimating assembly error for each species and then infers the final rate of gene family evolution based on these estimates. For the final set of genes estimated to be undergoing significant innovation in the branch leading to the collared kingfisher, we obtained functional annotations using InterProScan v. 5.47–82.0 (Jones *et al.* 2014) and performed enrichment analyses using both putative gene names in STRING (Szklarczyk *et al.* 2021) as well as GO terms in Revigo (http://revigo.irb.hr; accessed 2022 September 26).

## Results

### Genome assembly

**Fig. 1.**
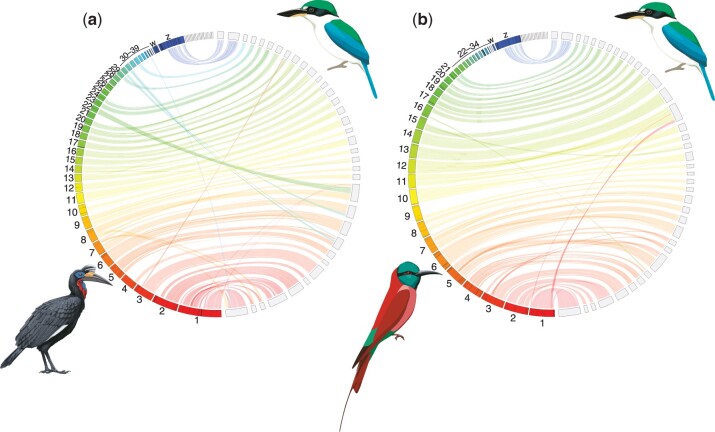
Genome assembly consistency plots between the collared kingfisher and (a) Abyssinian ground-hornbill and (b) northern carmine bee-eater. Unlabeled segments depict kingfisher scaffolds (right side of circle) comprising 75% of the genome, with regions closer than 100 kb considered contiguous. Labeled segments indicate chromosomes in the existing reference genomes. Note few inversions, near chromosome-level scaffold sizes, and generally one-to-one mapping between scaffolds and chromosomes.

We obtained a total of 68X read coverage (95.9 Gb total data) with paired-end 150-bp reads. The assembly comprised 15,043 scaffolds greater than 1 kb in size, with a final assembly size of 1.13 Gb and an estimated genome size of 1.40 Gb based on kmer analysis. The contig N50 was 155 kb and the scaffold N50 was 19 Mb. We found 96.6% complete single-copy genes relative to the BUSCO v. 5 aves_odb10 database [BUSCO score: C: 96.6% (S: 94.7%, D: 1.9%), F: 0.7%, M: 2.7%, *n*: 8,338], suggesting that the draft genome is mostly complete. Most scaffolds were highly contiguous and mapped to single chromosomes in related species (Fig. 1). In only a few cases did individual kingfisher scaffolds map to multiple chromosomes (Fig. 1).

### Population demographics

**Fig. 2.**
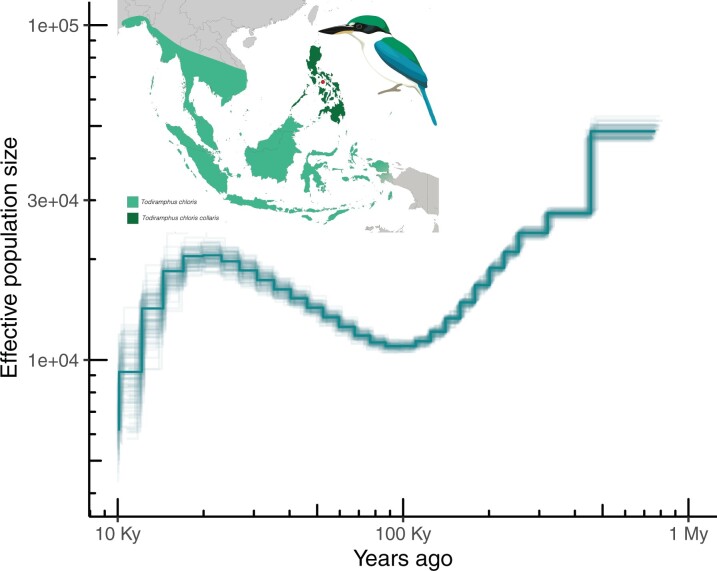
Population demographic history of the collared kingfisher inferred from PSMC analysis of the new draft genome. The bold line shows mean population sizes through time, and transparent lines depict uncertainty as 100 bootstraps based on resampling of genomic scaffolds. We used a generation time of 4.8 years and a mutation rate of 2.23 × 10^−8^ substitutions per generation (see *Materials and Methods* for details). Inset shows range of *Todiramphus chloris collaris* (dark green), along with sampling location of specimen (circle) and broader *T. chloris* species range (light green, range extends further west but is not shown).

We found fluctuations in effective population size (Ne) through the Quaternary Period, including a dip at 100 kya and a global maximum population size at ∼1 Mya. There was a substantial decline in Ne into the present (Fig. 2).

### Gene family evolution

**Fig. 3.**
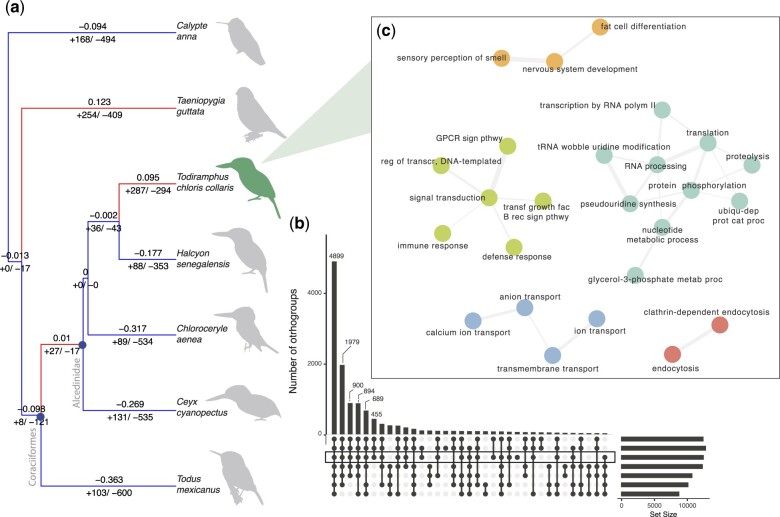
Genomic novelty in the collared kingfisher. a) Based on a total of 14325 gene families estimated with OrthoFinder, phylogeny shows the number of orthologous gene families significantly expanded (plus signs) and contracted (minus signs) for each branch (numbers below branches) using CAFE. Numbers below branches and branch colors correspond to direction (red: positive, blue: negative) of average net gain in number of genes per family. b) Upset plot showing overlap in orthogroups among species (vertical bars) and total orthogroups for each species (horizontal bars, species order the same as in a). For visualization purposes, overlaps <450 are not shown. c) Gene function network produced from 118 unique GO terms in Revigo for genes expanded in the collared kingfisher relative to its MRCA. Lines show semantic overlap among GO terms, and colors represent clusters of GO terms with similar gene functions.

OrthoFinder identified potential orthologous genes shared between the collared kingfisher and the other 6 bird genomes (Fig. 3b). Clustering of gene families revealed 14,325 shared orthogroups comprising 99,576 total genes (98.3% of the total 101,265 genes across the 7 genomes; 96.7% for the collared kingfisher only). We found that 4,899 orthogroups were present in all species and 259 were orthogroups present in only a single species. The collared kingfisher had 2.5% genes in unique, species-specific orthogroups (zebra finch was the highest at 3.8%). CAFE analysis identified gene family expansions (i.e. increases in the rates of gain of gene families per orthogroup) in hummingbird, finch, and collared kingfisher and contractions in the other species (Fig. 3a). In collared kingfisher, we found 287 new orthogroups (489 genes) and 294 lost orthogroups (352 genes) relative to the most recent common ancestor (MRCA) with the woodland kingfisher (*H. senegalensis*). GO term clustering analyses revealed that gene families significantly expanded in *Todiramphus* include those involved in immune response, metabolism, and signaling (Fig. 3c). The additional enriched protein terms in *Todiramphus* include collagen and ATP binding (see https://github.com/celiason/Todiramphus_Genome for STRING protein network). Two genes involved in bitter taste perception, *TAS2R7* and *TAS2R40*, are also estimated to be novel in the collared kingfisher relative to other species within its respective order, Coraciiformes.

## Discussion

We present a high-quality draft genome assembly for the collared kingfisher, an island-dwelling “great speciator” and member of a rapidly radiating genus of kingfishers. The assembly has high contig (N50 contig = 155 kb) and scaffold sizes (N50 scaffold = 19 Mb). Although the contigs are shorter owing to sequencing methodology (Illumina short-read sequencing vs PacBio long-read sequencing), the scaffold N50 meets the criteria for a “high-quality” reference genome according to the Vertebrate Genomes Project guidelines (https://vertebrategenomesproject.org; accessed 2022 September 26). The high BUSCO score (>96%) further suggests that the assembly is largely complete and on par with existing high-quality reference genomes (Feng *et al.* 2020)*. Todiramphus chloris* is one of the “great speciators” (Diamond and Mayr 1976), distributed widely across the Indo-Pacific with phenotypically differentiated and geographically separated forms. Although other avian clades have received greater attention, including *Zosterops* white-eyes (Cornetti *et al.* 2015; Gwee *et al.* 2020; Manthey *et al.* 2020) and Darwin’s Finches (Petren *et al.* 2005; Almén *et al.* 2016; Chaves *et al.* 2016), *Todiramphus* kingfishers could provide a distinct picture into avian diversification dynamics on islands due to evolutionary variation of dispersal abilities (e.g. migratory *Todiramphus sanctus* and sedentary *T. chloris*) and its phylogenetic position as a clade of nonpasserine birds. This genome thus sets the stage for future work on speciation mechanisms in the *Todiramphus* genus to give a broader understanding of avian speciation mechanisms on islands. For example, it will facilitate the study of chromosomal architecture evolution to test the hypothesis that the observed increased speciation rates in *Todiramphus* kingfishers (Andersen *et al.* 2018) are associated with chromosomal inversions.

The current population estimate for the *T. chloris* species complex based on statistical modeling is 8.8 million individuals (Callaghan *et al.* 2021), with a minimum estimate of 91k individuals. Our estimate of 50k individuals could differ because we are estimating effective population sizes (Ne) rather than absolute population size, or because we focus on an island subspecies within *Todiramphus chloris* rather than the whole species complex that is more broadly distributed across southeast Asia to eastern New Guinea (Fig. 2, Andersen *et al.* 2015). Other kingfisher species endemic to the Philippine Archipelago have estimated population sizes ranging from 63k (*Ceyx argentatus*) to ∼100k (106k for *Ceyx melanurus*, 109k for *Todiramphus winchelli*) (Callaghan *et al.* 2021). *Todiramphus chloris collaris* is estimated to have split from other *T. chloris* species around ∼100 kya (Andersen *et al.* 2015). Therefore, the fluctuations in Ne shown in our PSMC result after this time are expected to be unique to this lineage, whereas fluctuations prior to this divergence are expected to track one another (Cahill *et al.* 2016). Indeed, divergence time estimates for crown *Todiramphus* are <1 Mya (McCullough *et al.* 2019), and the elevated population sizes inferred ∼1 Mya likely reflect the widespread distribution of the *T. chloris* species complex (Andersen *et al.* 2015). Nadachowska-Brzyska *et al.* (2015) found a general pattern of decreases in Ne at the start of the last glacial period ∼115 kya across all birds. The pattern we find in the collared kingfisher differs in that we show an expansion in population size starting ∼100 kya (Fig. 2). This difference could be due either to difficulties with estimating population sizes from genomic data (e.g. stemming from difficulties with parameter selection in PSMC analyses, including mutation rates and generation times, or conflation of population size with historical changes in population structure) (Mather *et al.* 2020), increased land area on islands during glacial maxima (Heaney 1985), or that *T. chloris collaris* speciation in the Philippine Archipelago is less affected by paleoclimatic changes than other bird species. The changes in climate rather than just land bridges/connectivity through the Pleistocene/late Pliocene have recently been associated with allopatric splits between Philippine bird species (Hosner *et al.* 2014), and others have found similar results in Philippine rodents (Kyriazis *et al.* 2017). We anticipate that these results and further sampling of genome-inferred population demographics within *Todiramphus* will continue to inform and refine our understanding of avian diversification in the Philippines.

Given the locomotor, reproductive, and sensory challenges associated with navigating and surviving on islands, innovation in sensory genes might be critical to insular taxa. We found an expansion in the number of genes per gene family in *Todiramphus* relative to other kingfisher and coraciiform lineages (Fig. 3a). This was irrespective of variation in assembly quality (e.g. see Supplementary Table 2), as we accounted for this variation in the CAFE program (Han *et al.* 2013). Gene families that were expanded in *Todiramphus* include those involved in ion transport, immune system function, metabolism, immune system function, and signaling (Fig. 3c). Immune system genes are known to evolve rapidly across birds (Shultz and Sackton 2019). Novel pathogens on islands might select for attenuated immune response (Matson 2006) and differences in immune system genes compared to mainland species in birds (Armstrong *et al.* 2018). In addition to novel pathogen landscapes, climate unpredictability and limited ability to migrate off of islands during food shortages have been proposed to explain the reduced metabolic rates in insular birds (Noakes *et al.* 2013). This could potentially explain the expansion in metabolic genes in the collared kingfisher (Fig. 3). Dietary diversity is a hallmark of kingfishers, with diets and foraging modes ranging from gleaning for insects, to digging for worms and plunge-diving for fish (Woodall 1991; Fry 1992). Thus, it is not surprising that we find significant expansion in the G protein-coupled receptor family (Fig. 3c), specifically genes involved in vision (OPN1LW), olfaction (*OR5AN1*, *OR14I1*, *OR10AG1*, *OR14J1*), and taste perception (*TAS2R7*, *TAS2R40*; see https://github.com/celiason/Todiramphus_Genome for annotations). Taste perception in birds has been underappreciated, but recent work on evolutionary shifts in taste receptor genes (Baldwin *et al.* 2014; Zhao *et al.* 2015) hints at their biological relevance. Taken together, our results highlight potential targets for further work in functional and comparative genomics on the sensory abilities and immune system function of *Todiramphus* kingfishers. Expanding our comparative genomics approach to closely related *Todiramphus* and other kingfisher species could shed light on genetic mechanisms of evolutionary change associated with island living.

## Supplementary Material

jkac260_Supplementary_Data

## Data Availability

All data for this project are available publicly at NCBI under BioProject Accession PRJNA851081, including raw Illumina reads (SRA SRR21604398) and the genome contig assembly (WGS JANVCC000000000). All scripts for genome analyses are publicly available at https://github.com/celiason/Todiramphus_Genome. Supplemental material is available at G3 online.
