# Supplementary material for: Genomic novelty within a “great speciator” revealed by a high-quality reference genome of the collared kingfisher (Todiramphus chloris collaris)
Source: G3 (Bethesda). 2022 Sep 26;12(11):jkac260. doi: 10.1093/g3journal/jkac260 (PMC9635628; doi:10.1093/g3journal/jkac260)
Supplement: jkac260_Supplementary_Data [file jkac260_supplementary_data.pdf]

Supplementary Tables

Table S1. Genomic data sources and analyses for which they were used. Annot: genome annotation, Jup: assembly consistency plots produced with JupiterPlot, CAFE: Orthofinder and CAFE analysis of gene family evolution.

Species	GB Accession	Citation	Annot	Jup	CAFE
Zebra finch<br>*Taeniopygia guttata*	GCF_008822105.2	Rhie et al. (2021)	x		x
Anna's hummingbird<br>*Calypte anna*	GCF_003957555.1	Rhie et al. (2021)	x		x
Woodland kingfisher<br>*Halcyon senegalensis*	GCA_013397595.1	Unpublished			x
Red jungle fowl<br>*Gallus gallus*	GCA_000002315.5	International Chicken Genome Sequencing Consortium (2004)	x		
Budgerigar<br>*Melopsittacus undulatus*	GCA_012275295.1	Unpublished	x		
Golden eagle<br>*Aquila chrysaetos*	GCA_900496995.4	Unpublished	x		
American pygmy kingfisher<br>*Chloroceryle aenea*	GCA_013399075.1	Unpublished			x
Indigo-banded kingfisher<br>*Ceyx cyanopectus*	GCA_013401355.1	Unpublished			x
Puerto Rican tody<br>*Todus mexicanus*	GCA_013389965.1	Unpublished			x
Abyssinian ground-hornbill<br>*Bucorvus abyssinicus*	GCA_009769605.1	Unpublished		x	
Northern carmine bee-eater<br>*Merops nubicus*	GCA_009819595.1	Unpublished		x	

Table S2. Estimated assembly errors for CAFE gene family evolution models. BUSCO scores calculated with BUSCO v. 5 using the aves_odb10 database.

Species	BUSCO score (%)	Annotated genes	CAFE error
Taeniopygia guttata	98.8	19236	0.000
Calypte anna	97.9	17137	0.000
Ceyx cyanopectus	54.2	12043	0.178
Chloroceryle aenea	73.8	12200	0.034
Halcyon senegalensis	80.1	14274	0.007
Todiramphus chloris collaris	93.0	21562	0.158
Todus mexicanus	39.4	10073	0.240

Supplementary References

International Chicken Genome Sequencing Consortium, 2004 Sequence and comparative analysis of the chicken genome provide unique perspectives on vertebrate evolution. Nature 432: 695–716.

Rhie, A., A. Meyer, A. Kautt, P. Franchini, R. H. S. Kraus *et al.*, 2021 *Towards Complete and Error-free Genome Assemblies of All Vertebrate Species*.
